# Composition and Bioactivity of Essential Oil from *Citrus grandis* (L.) Osbeck ‘Mato Peiyu’ Leaf

**DOI:** 10.3390/molecules22122154

**Published:** 2017-12-05

**Authors:** Mei-Lin Tsai, Cai-Di Lin, Keh Ai Khoo, Mei-Ying Wang, Tsang-Kuei Kuan, Wei-Chao Lin, Ya-Nan Zhang, Ya-Ying Wang

**Affiliations:** 1 Department of Cosmetic Science, Chia Nan University of Pharmacy & Science, Tainan 71710, Taiwan; lin820417@gmail.com (C.-D.L.); meiying@mail.cnu.edu.tw (M.-Y.W.); weilin@mail.cnu.edu.tw (W.-C.L.); 2 School of Pharmaceutical Sciences, University of Science Malaysia, Penang 11800, Malaysia; kehai736@gmail.com; 3 Department of Chemical Science, National Chung Hsing University, Taichung 40227, Taiwan; kuan@getech.com.tw; 4 Department of Pharmacy, Xiamen Medical University, Xiamen 361023, China; zyn@xmmc.edu.cn (Y.-N.Z.); wyy@xmmc.edu.cn (Y.-Y.W.); 5 Fujian Provincial Key Laboratory of Genuine Medicinal Materials Bio-Engineering, Xiamen 361023, China

**Keywords:** essential oil, chemical composition, antimicrobial, antioxidant, anti-inflammation, antityrosinase

## Abstract

‘Mato Peiyu’ pomelo (*Citrus grandis* (L.) Osbeck ‘Mato Peiyu’) leaves from pruning are currently an agricultural waste. The aim of this study was to isolate essential oils from these leaves through steam distillation (SD) and solvent-free microwave extraction (SFME) and to evaluate their applicability to skin care by analyzing their antimicrobial, antioxidant (diphenyl-1-picrylhydrazyl scavenging assay, β-carotene/linoleic acid assay, and nitric oxide scavenging assay), anti-inflammatory (5-lipoxygenase inhibition assay), and antityrosinase activities. The gas chromatography–mass spectrometry results indicated that the main components of ‘Mato Peiyu’ leaf essential oils were citronellal and citronellol, with a total percentage of 50.71% and 59.82% for SD and SFME, respectively. The highest bioactivity among all assays was obtained for 5-lipoxygenase inhibition, with an IC_50_ value of 0.034% (*v*/*v*). The MIC_90_ of the antimicrobial activity of essential oils against *Escherichia coli*, *Pseudomonas aeruginosa*, *Staphylococcus aureus*, and *Candida albicans* ranged from 0.086% to 0.121% (*v*/*v*). Citronellal and citronellol were the main contributors, accounting for at least 54.58% of the essential oil’s bioactivity. This paper is the first to report the compositions and bioactivities of ‘Mato Peiyu’ leaf essential oil, and the results imply that the pomelo leaf essential oil may be applied in skin care.

## 1. Introduction

The pomelo (*Citrus grandis* (L.) Osbeck), belonging to the Rutaceae family, is a tropical and subtropical fruit. In some places, pomelo is not only a fruit but is also used in folk medicine against fatigue, loss of energy, lack of vitality, bruising, wounds, acne, or mild skin disorders [1]. Several varieties of pomelo are present in Taiwan. ‘Mato Peiyu’ (*Citrus grandis* (L.) Osbeck ‘Mato Peiyu’) has 180 years of cultural history and is principally distributed in Southern Taiwan. ‘Mato Peiyu’ is the most widely consumed variety of the large pomelo, with an agreeable taste because of its soft juicy pulp and a pleasant sugar to acid ratio [2].

To produce superior quality fruit, the ‘Mato Peiyu’ tree must be pruned. Pruning results in many unused leaves and branches becoming agricultural waste. In Taiwanese folk culture, bathing with pomelo leaves on Chinese New Year’s Eve was believed to ward off bad luck and prevent illness. Pomelo leaves exhibit a mind calming effect. Additionally, pomelo-leaf baths help dredge the meridian and promote blood circulation [3]. Drinking boiled pomelo leaf water was a traditional therapy to treat headache and stomach pain. If the bioactivities of pomelo leaves could be identified, this agricultural waste can be recycled and reused, thereby increasing the commercial value of local crops.

Essential oils are volatile compounds extracted from the leaves, flowers, fruits, stalks, roots, and resins of plants and are widely used in aromatherapy, perfumes, cosmetics, and the food industry [4]. The pomelo is a large fruit of the genus *Citrus*. Citrus essential oil is one of the most widely used essential oils because of its characteristic fresh sweet scent. Lemon (*Citrus limon*), orange (*Citrus sinensis*), and bergamot (*Citrus aurantium*) have been commonly applied in aromatherapy to relieve stress, anxiety, and depression [5,6]. In a pentobarbital sleeping-time model of Swiss male mice, *Citrus aurantium* could reduce insomnia [7].

Additionally, citrus essential oils have proven antimicrobial [8,9,10,11], antioxidant [12,13,14], anti-inflammatory [15], antipigmentation [16], nematocidal [17], and anticarcinogenic abilities [18]. Studies have revealed that pomelo peel essential oils had a DPPH scavenging effect [19] and exhibited broad-spectrum antimicrobial activity against *Penicillium chrysogenum*, *Bacillus subtilis*, *Staphylococcus aureus*, *Escherichia coli* [20], and *P. expansum* [21]. Additionally, essential oils from the pomelo leaf were demonstrated to possess antidermatophytic potential against *Microsporum* and *Trichophyton* species [22]. Aumeeruddy-Elalfi et al. reported that pomelo leaf essential oil had the strongest antityrosinase capacity among 19 traditional medicinal plants in Mauritius [1], and could be used to inhibit the formation of melanin pigment [23,24]. Therefore, pomelo essential oil possesses multiple functions in common with other citrus plants. However, the bioactivities and composition of essential oil from the ‘Mato Peiyu’ pomelo leaf have not yet been investigated.

Essential oils have been used as an alternative therapy for skin problems, such as the application of tea tree oil to treat acne vulgaris and dandruff [25]. Skin inflammation allows penetration of irritants and allergens and predisposes patients to colonization and infection by microbial organisms [26]. Additionally, inflammation induces skin hyperpigmentation by enhancing tyrosinase activity and melanogenesis [23,24,27]. Moreover, oxidative stress and altered antioxidant defenses play some role in the pathogenesis of dermatitis, and antioxidants might be beneficial in dermatitis treatment [28,29]. It implies that skin care products for people with inflammatory skin problems should possess anti-inflammatory, antimicrobial, antioxidant, and antipigmentary properties.

This study analyzed the chemical profile and bioactivities of ‘Mato Peiyu’ leaf essential oil, including antimicrobial, antioxidant, anti-inflammatory, and antipigmentary abilities to evaluate its potential application in skin care. The oil was extracted through conventional steam distillation (SD) and solvent-free microwave extraction (SFME), and its components were determined through gas chromatography-mass spectrometry (GC-MS). To clarify the contributions of the main components to the bioactivity of essential oils, their bioactivities of the main components were also be determined using the same method.

## 2. Results and Discussion

### 2.1. Yields of Essential Oils

The isolation method affects the constituents of essential oils, thereby influencing the efficiency of biological activities. SFME isolates and concentrates essential oil from the plant materials through a combination of microwave heating and dry distillation at atmospheric pressure. This technique provides savings in costs, particularly energy and time [30]. Hence, in addition to conventional SD, SFME was employed to extract the essential oils. The results show that the yields of ‘Mato Peiyu’ leaf essential oils obtained through SFME and SD were similar (0.15% and 0.13%, respectively), although the extraction time through SFME was shorter than that through SD (40 min vs. 240 min). The relatively low yields were similar to those of Tunisian pomelo leaf essential oil extracted through hydrodistillation (HD) (0.15%) [31]. Some studies have reported that novel microwaved-assisted heating methods (microwave-assisted HD and SFME) resulted in a larger yield than did the conventional methods (HD and SD) for extracts such as guava (*Psidium guajava*) [32] and lavender (*Lavandula hybrida*) essential oils [33]; however, other studies have shown no differences between these methods. Yields of *Rosmarinus officinalis*, *Ocimum basilicum*, *Mentha crispa*, and *Thymus vulgaris* essential oils isolated through SFME and HD were quantitatively similar [30,34,35]. These results suggest that whether SFME or SD produces a higher yield of essential oils depends on the plant species; however, SFME is more time and energy efficient than SD [30,32].

### 2.2. Composition of Essential Oils

GC–MS analyses of the ‘Mato Peiyu’ leaf essential oils revealed that 26 and 21 compounds constituted 98.89% and 96.81% of the total integrated GC peak area of each essential oil obtained through SD and SFME, respectively (Table 1). The most abundant compound group in SD essential oil (SD-EO) was aldehydes (34.54%), primarily monoterpene aldehydes. Alcohols were the second largest compound group (19.26%), primarily monoterpene alcohols, which constituted 17.17% of the essential oil, followed by diterpene alcohols (1.65%) and sesquiterpene alcohols (0.44%). Other compound groups in the SD-EO were monoterpenes (18.34%), sesquiterpenes (15.78%), monoterpene esters (10.00%), sesquiterpene epoxides (0.88%), and furans (0.09%). Similarly, the most abundant compound group identified in SFME essential oil (SFME-EO) was aldehydes (30.98%), mainly monoterpene aldehydes (30.87%) with a trace quantity of non-terpene aldehydes (0.11%). The second largest compound group was alcohols (30.64%), of which monoterpene alcohols were the principal group, comprising 29.20% of the essential oil, followed by sesquiterpene alcohols (1.02%) and diterpene alcohols (0.42%). Monoterpenes (22.61%), monoterpene esters (5.70%), sesquiterpenes (4.23%), sesquiterpene epoxides (2.21%), furans (0.28%), and monoterpene cyclic ethers (0.16%) were also identified in the SFME-EO. In comparison with the SFME-EO, fewer alcohols but more sesquiterpenes and aldehydes were present in the SD-EO. Moreover, the oxygenated monoterpene fractions in the SD-EO and SFME-EO were similar (61.71% and 65.93%, respectively) (Table 2).

The major constituents of the SD-EO were citronellal (34.54%), citronellol (16.17%), citronellyl butyrate (10.00%), β-caryophyllene (8.22%), and (−)-β-pinene (7.33%). By contrast, the main components of the SFME-EO were citronellal (30.87%), citronellol (28.95%), (−)-β-pinene (9.58%), α-ocimene (6.38%), and citronellyl butyrate (5.70%) (Table 1). Citronellal, which was the most abundant compound in both essential oils, belongs to the family of monoterpene aldehydes. Citronellol, a monoterpene alcohol, was identified as the second major compound in the essential oils and was of a higher relative percentage in the SFME-EO compared with the SD-EO. More citronellyl butyrate and β-caryophyllene were found in the SD-EO than in the SFME-EO. Moreover, the constituents of minor components recovered using these two methods also differed. No furocoumarin (a phototoxic ingredient) was found in either the SD-EO or SFME-EO, although ‘Mato Peiyu’ belongs to the citrus genus (Table 1).

Lin et al. determined the two most abundant volatile components in the fresh leaves of the ‘Wendun’ pomelo in Taiwan to be citronellal and citronellol, which are identical to those in ‘Mato Peiyu’ leaf essential oil. However, the relative percentage of citronellal (54.26%) and citronellol (11.68%) were notably different from those of ‘Mato Peiyu’. Moreover, the other main compounds found in the ‘Wendun’ pomelo (isopulegol,1,3,8-*p*-menthatriene and spiro[2.5]octane) also differed [36]. Studies have reported the principal constituents of pomelo leaf essential oil to be E-ocimene and β-pinene in China [37], limonene, linalool, and citronellal in Iran [38], and spathulenol and β-caryophyllene in Uttarakhand [20], respectively. These analyses indicate that the constituents and their relative percentage in pomelo leaf essential oil vary considerably depending on genetic and geographical factors.

### 2.3. Antimicrobial Activity of Essential Oils and the Main Components

*S. aureus*, *Pseudomonas aeruginosa*, *E. coli*, and *Candida albicans* are clinically relevant skin and wound pathogens [39]. *S. aureus* is the most common organism causing bacterial infection in patients with atopic dermatitis [40]. *S. aureus*, *P. aeruginosa*, and *E. coli* are the main causes of infection and delayed healing in acute, chronic, and postoperative wounds [41,42,43]. Meanwhile, *C. albicans* is the principal infectious agent in cutaneous and nail candidosis and invasive fungal infections in hospitalized patients [44,45]. These four pathogens have also been reported in contaminated skin care products [46,47,48]. Hence, the antimicrobial properties of essential oils were determined against *S. aureus*, *P. aeruginosa*, *E. coli*, and *C. albicans*. The results show that ‘Mato Peiyu’ leaf EO exhibited effective antimicrobial activities against four tested pathogens (Table 3). Saeb et al. observed that pomelo leaf essential oil possessed greater antibacterial activity against *E. coli* and *S. aureus* than other citrus (*C. reticulata* and *C. limon*) leaf essential oils from Iran [49]. ‘Mato Peiyu’ leaf SD-EO exhibited the strongest antimicrobial effect against *S. aureus*, followed by *E. coli*, *P. aeruginosa*, and *C. albicans*, with MIC_90_ values of 0.086%, 0.090%, 0.103% and 0.116% (*v*/*v*), respectively. By contrast, the SFME-EO was most potent against *P. aeruginosa*, followed by *S. aureus*, *E. coli*, and *C. albicans*; MIC_90_ values were 0.088%, 0.098%, 0.116% and 0.121% (*v*/*v*), respectively. In contrast to the SFME-EO, the SD-EO exhibited larger microbial inhibitory effects against all pathogens, except *P. aeruginosa* (Table 3).

**Figure 1:**
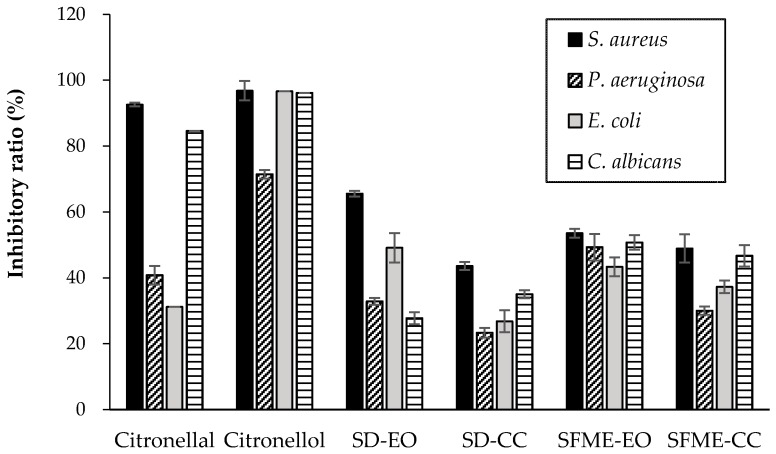
Inhibitory ratio (%) of ‘Mato Peiyu’ leaf essential oils (SD-EO and SFME-EO), citronellal, and citronellol against four microbial strains. SD-CC and SFME-CC were the mixtures of citronellal and citronellol, which were tested at concentrations determined according to composition ratios relative to the essential oils obtained through SD and SFME, respectively. SD-EO, SFME-EO, citronellal, and citronellol were measured at a concentration of 0.0500% (*v*/*v*). SD-CC and SFME-CC were measured at concentrations of 0.0254% and 0.0299% (*v*/*v*), respectively. Data are presented as the mean ± standard deviation based on triplicate determination.

Citronellal and citronellol were the most abundant constituents of ‘Mato Peiyu’ leaf essential oils (Table 1), at 51.71% and 59.82% in the SD-EO and SFME-EO, respectively. Therefore, the microbial inhibitory ratio of these two compounds were evaluated to study their contributions to the antimicrobial capabilities of essential oils. The results showed that at a concentration of 0.05% (*v*/*v*), citronellal exhibited the strongest antimicrobial effect against *S. aureus*, at an inhibitory ratio of up to 92.60%, followed by the antimicrobial effect against *C. albicans* (84.58%), and relatively weaker inhibitory effects against the Gram negative bacteria *P. aeruginosa* (40.78%) and *E. coli* (31.19%). By contrast, citronellol exhibited marked antimicrobial effects against *S. aureus*, *E. coli*, and *C. albicans*, all at a 96% inhibitory ratio, whereas a weaker antimicrobial effect was recorded against *P. aeruginosa* (71.44%) (Figure 1). The results demonstrated that citronellol was more potent than citronellal against all bacteria except *S. aureus*, against which the inhibitory ability of the two constituents was similar. Citronellol also exhibited greater antimicrobial activity compared with the SD-EO and SFME-EO, indicating that citronellol is the stronger antimicrobial component in ‘Mato Peiyu’ essential oil. Studies have observed that citronellol and citronellal were more potent against *S. aureus* than against *E. coli* [50,51,52]. The antimicrobial potency of essential oils against bacteria depends on the structure of the microbial cell membrane [53]. Essential oils inhibit bacterial growth through binding of the constituent molecules to the bacterial wall, which alters the bacterial cell membrane properties (hydrophobicity, surface charge, and membrane integrity), leading to leakage of intracellular constituents and cell lysis [54]. Furthermore, the ability of microorganisms to metabolize the compounds affects the potency of essential oil in inhibiting bacterial growth [55].

This study was extended to determine the microbial inhibitory ratios of citronellol and citronellal mixtures (SD-CC and SFME-CC) and to evaluate their antimicrobial contribution in EO. The contributions of the antimicrobial activity of the mixtures to that of the essential oils were calculated according to the results in Figure 1 and are presented in Table 4. The contributions of the SD-CC to activity against *S. aureus* and *P. aeruginosa* were 66.46% and 70.86%, respectively, indicating that the antimicrobial capability of the SD-EO against *S. aureus* and *P. aeruginosa* was derived mainly from citronellal and citronellol, but minor compounds also provided some contribution. Furthermore, the contribution of the SD-CC to activity against *E. coli* was 54.58%, suggesting that minor components in the essential oil had a similar influence on the inhibitory ratio. Unexpectedly, the contribution of the SD-CC to activity against *C. albicans* was notably high (126.17%), indicating that other constituents in the essential oil might have attenuated the antimicrobial activity of citronellal and citronellol against *C. albicans*. By contrast, the contributions (%) of the SFME-CC to activity against *S. aureus*, *E. coli*, and *C. albicans* were 91.37%, 85.91%, and 91.98%, respectively, suggesting that microbial growth inhibition was mostly attributable to citronellal and citronellol, whereas the contributions of other compounds were smaller. The contribution of the SFME-CC to activity against *P. aeruginosa* was 60.89%, indicating that minor components contributed a similar effect to the antibacterial ratio. This antimicrobial behavior could be explained by the complex interaction between constituents in the essential oils that produced either synergistic or antagonistic effects on microbial inhibition [56].

The isolation method influences the efficiency of antimicrobial activity. In addition to citronellal and citronellol, the other minor components of SD-EO also demonstrated superior antimicrobial activity against *S. aureus* and *E. coli* than SFME-EO did (Figure 1). The contributions of the minor components were 33.54% and 45.42%, respectively. By contrast, the antimicrobial activity of SFME-EO was mostly from citronellal and citronellol, and the other minor components only contributed 8.63% and 14.09% for *S. aureus* and *E. coli*, respectively (Table 4). Moreover, previous studies have revealed that sesquiterpene showed superior antibacterial activities against *S. aureus* and *E. coli* [57,58,59]. SD-EO comprised more sesquiterpene than did SFME-EO (Table 2). Therefore, SD-EO exhibited higher anti-*S. aureus* and anti-*E. coli* activity than SFME-EO did (Table 3). SFME-CC exhibited a higher inhibitory ratio against *P. aeruginosa* than did SD-CC with 30.01% and 23.30%, respectively (Figure 1). In addition, the other minor components of SFME-EO demonstrated higher anti-*P. aeruginosa* activity than that of SD-EO, with 39.11% and 29.14% contributions, respectively. This resulted in SFME-EO exhibiting superior antimicrobial capacity against *P. aeruginosa* compared with SD-EO.

### 2.4. Antioxidant Activity of Essential Oils and the Main Components

‘Mato Peiyu’ leaf essential oils were evaluated for their antioxidant activities through a 2,2-diphenyl-1-picrylhydrazyl (DPPH) radical scavenging assay, β-carotene/linoleic acid assay, and nitric oxide (NO) radical scavenging assay. The results are reported in Table 5. The SD-EO was a stronger DPPH radical scavenger with an SC_50_ value of 0.752% (*v*/*v*) compared with the SFME-EO (0.876%, *v*/*v*). Several studies have demonstrated that citrus leaf essential oils were stronger DPPH radical scavengers than were citrus peel essential oils [19,60,61]. Additionally, citrus leaf essential oil exhibited a stronger DPPH scavenging ability (SC_50_ values of 2.1 ± 0.23%) than did widely used and commercially available herb essential oils, including lavender, peppermint, rosemary, lemon, grapefruit, and frankincense [62]. The DPPH scavenging capacity of the Mato Peiyu leaf essential oils was lower than that of α-tocopherol in this study (Table 5), but these essential oils scavenged DPPH radicals more than other plant essential oils did [19,60,61,62].

In the β-carotene/linoleic acid test, the SFME-EO exhibited a notably larger antioxidant capacity compared with the SD-EO, with IC_50_ values of 0.086% and 0.147% (*v*/*v*), respectively, but it was slightly smaller than that of α-tocopherol (IC_50_ value of 0.060% (*v*/*v*)). By contrast, no differences (*p* > 0.05) were observed between the SD-EO and SFME-EO in NO radical scavenging activity, which was equivalent to one third of that of α-tocopherol. The influence of the extraction methods, SD and SFME, on the antioxidant effect of ‘Mato Peiyu’ essential oil was inconsistent among the three assays. Nevertheless, the SD-EO or SFME-EO exhibited the strongest antioxidant properties in the β-carotene/linoleic acid assay, followed by the NO radical scavenging assay. Both oils were weak DPPH radical scavengers (Table 5). Previous research demonstrated that essential oils from *Cymbopogon martinii*, *T. vulgaris*, *Lindernia anagallis*, *Pelargonium* × *fragrans*, and *Melaleuca alternifolia* exhibited stronger antioxidant capacity in the β-carotene/linoleic acid assay compared with NO and DPPH radical scavenging assays [63].

**Figure 2:**
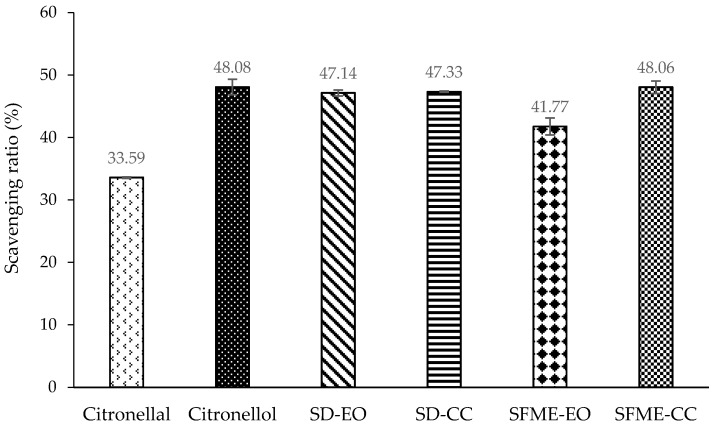
Diphenyl-1-picrylhydrazyl (DPPH) radical scavenging ratio (%) of ‘Mato Peiyu’ leaf essential oils, citronellal, and citronellol. SD-EO, SFME-EO, citronellal, and citronellol were measured at a concentration of 0.500% (*v*/*v*). SD-CC and SFME-CC were measured at concentrations of 0.254% and 0.299% (*v*/*v*) according to the composition ratios in 0.500% (*v*/*v*) SD-EO and SFME-EO, respectively. Data are presented as the mean ± standard deviation based on triplicate determination.

**Figure 3:**
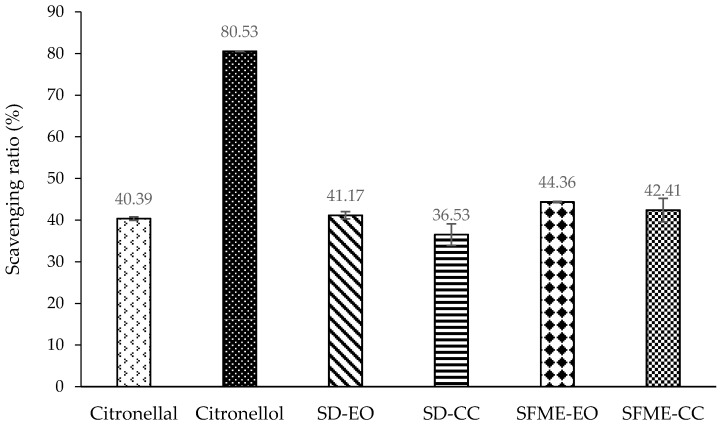
Nitric oxide (NO) radical scavenging ratio (%) of ‘Mato Peiyu’ leaf essential oils, citronellal, and citronellol. SD-EO, SFME-EO, citronellal, and citronellol were measured at a concentration of 0.250% (*v*/*v*). SD-CC and SFME-CC were measured at concentrations of 0.127% and 0.150% (*v*/*v*) according to the composition ratios in 0.250% (*v*/*v*) SD-EO and SFME-EO, respectively. Data are presented as the mean ± standard deviation based on triplicate determination.

**Figure 4:**
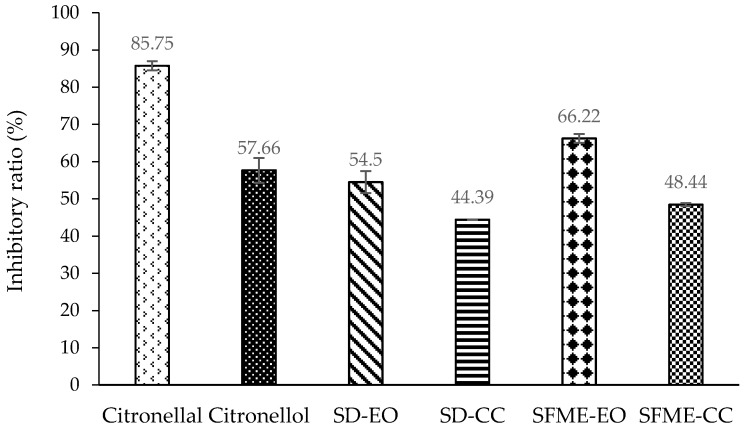
β-Carotene/linoleic acid inhibitory ratio (%) of ‘Mato Peiyu’ leaf essential oils, citronellal, and citronellol. SD-EO, SFME-EO, citronellal, and citronellol were measured at a concentration of 0.150% (*v*/*v*). SD-CC and SFME-CC were measured at concentrations of 0.076% and 0.090% (*v*/*v*) according to the composition ratios in 0.150% (*v*/*v*) SD-EO and SFME-EO, respectively. Data are presented as the mean ± standard deviation based on triplicate determination.

As in the antibacterial assay, the bioactivities of citronellal, citronellol, and a mixture containing both were also measured. At a concentration of 0.500% (*v*/*v*), citronellol had stronger DPPH radical scavenging activity than did citronellal. The scavenging ratios were 48.08% and 33.59%, respectively (Figure 2). This behavior is consistent with the finding by Singh et al. [64]. In addition, citronellol was a more effective NO radical scavenger than citronellal at a concentration of 0.250% (*v*/*v*). The scavenging ratios were 80.53% and 40.39% for citronellol and citronellal, respectively (Figure 3). However, citronellol exhibited a smaller inhibitory effect against β-carotene/linoleic acid than that of citronellal at a concentration of 0.150% (*v*/*v*). The inhibitory ratios were 57.66% and 85.75% for citronellol and citronellal, respectively (Figure 4). Among the three antioxidant assays, citronellol had a stronger effect in NO scavenging and β-carotene/linoleic acid inhibition than it did in DPPH radical scavenging. By contrast, citronellal exhibited higher antioxidant activity in the β-carotene/linoleic acid test and the lowest in the DPPH assay (Figure 2, Figure 3 and Figure 4).

The antioxidant activity and contributions of the SD-CC and SFME-CC are shown in Figure 2, Figure 3 and Figure 4 and Table 4. The DPPH radical scavenging ability of the SD-EO was almost entirely attributable to the citronellal and citronellol mixtures, with the contribution of the SD-CC being approximately 100%. By contrast, the contribution of the SFME-CC exceeded 100%, indicating interference in DPPH radical scavenging by other components in the SFME-EO. The interference might contribute to SFME-EO exhibiting a lower DPPH scavenging capacity than SD-EO.

The antioxidant capacity of the SD-EO in the NO assay was mostly attributable to the SD-CC, with a contribution of 88.73%. By contrast, the NO scavenging activity of the SFME-EO was almost entirely attributable to the SFME-CC (95.60% contribution). However, other constituents of the essential oil might have enhanced the inhibitory capabilities of the SD-EO and SFME-EO against β-carotene/linoleic acid on the basis of the contributions of the SD-CC and SFME-CC (81.45% and 73.15%, respectively) (Table 4). Therefore, in addition to the primary components exhibiting their greatest antioxidant activity against β-carotene/linoleic acid (Figure 4), the other constituents of the essential oils exhibited stronger antioxidant activity in the β-carotene/linoleic acid assay than they did in the DPPH and NO scavenging tests, which contributed to the essential oils having the greatest activity in that assay (Table 5). SFME-CC exhibited a higher inhibitory ratio than SD-CC with 48.44% and 44.39%, respectively (Figure 4). In addition, the other minor components of SFME-EO achieved a higher β-carotene/linoleic acid inhibitory activity than that of SD-EO, with 26.85% and 18.55% contributions, respectively. These resulted in SFME-EO exhibiting superior anti-β-carotene/linoleic acid oxidation compared with SD-EO.

### 2.5. Anti-Inflammatory Activity of Essential Oils and the Main Components

**Figure 5:**
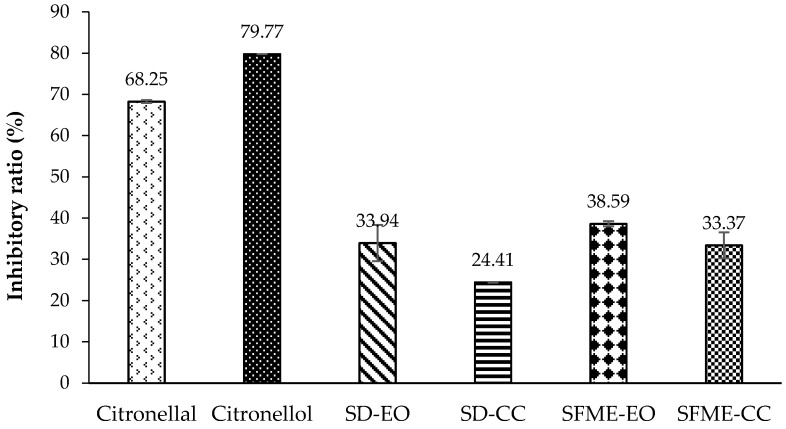
5-Lipoxygenase (5-LOX) inhibitory ratio (%) of ‘Mato Peiyu’ leaf essential oils, citronellal, and citronellol. SD-EO, SFME-EO, citronellal, and citronellol were measured at a concentration of 0.0200% (*v*/*v*). SD-CC and SFME-CC were measured at concentrations of 0.0101% and 0.0120% (*v*/*v*) according to the composition ratios in 0.0200% (*v*/*v*) SD-EO and SFME-EO, respectively. Data are presented as the mean ± standard deviation based on triplicate determination.

The 5-lipoxygenase (5-LOX) inhibitory activity of ‘Mato Peiyu’ leaf EO was similar between SD and SFME. Both SD-EO and SFME-EO exhibited stronger activity in the 5-LOX assay than they did in the antioxidant assays, with IC_50_ values of 0.039% and 0.034% (*v*/*v*), respectively (Table 5). This phenomenon was also observed in citronellal and citronellol (Figure 2, Figure 3, Figure 4 and Figure 5). Additionally, citronellol was a stronger 5-LOX inhibitor than was citronellal, with inhibitory ratios of 79.77% and 68.25% (*v*/*v*), respectively, at a concentration of 0.020% (Figure 5). The 5-LOX inhibitory ratios of the SD-CC and SFME-CC are shown in Figure 5. The contributions of the SD-CC and SFME-CC to the 5-LOX inhibitory activity of the essential oils were 71.92% and 86.47%, respectively (Table 4). These results imply that the potent 5-LOX inhibition of the Mato Peiyu leaf essential oil was attributable the combined effects of the main components and the minor constituents, in which the major contributors were citronellal and citronellol.

Arachidonic acid (AA) is released from cell membranes in response to various stimuli. AA can be metabolized by two major enzymatic pathways: cyclooxygenase (COX) and 5-LOX, leading to the production of proinflammatory mediators, prostanoids, and leukotrienes [65]. NO is a free oxygen radical and can act as a cytotoxic agent in pathological processes, particularly in inflammatory disorders [66,67,68,69,70]. Therefore, the inhibition of COX, 5-LOX, and NO production are used to evaluate the anti-inflammatory activity of a substrate.

Studies have determined that citronellol inhibited NO and PGE2 production in a concentration-dependent manner by inhibiting iNOS enzymatic activity and reducing the LPS-induced COX-2 protein and mRNA expression levels [71,72]. Additionally, intraperitoneal administration of citronellal suppressed carrageenan-induced mouse leukocyte migration to the peritoneal cavity and rat hind paw edema [73]. These results indicate that both citronellol and citronellal have substantial anti-inflammatory activity. The present study revealed that citronellol and citronellal accounted for a high percentage in the ‘Mato Peiyu’ leaf essential oil (50–60%) (Figure 1) and exhibited strong 5-LOX inhibition and NO scavenging activities (Table 5). Therefore, ‘Mato Peiyu’ leaf has potential for treating inflammation-associated disorders.

### 2.6. Antityrosinase Activity of Essential Oils and the Main Components

**Figure 6:**
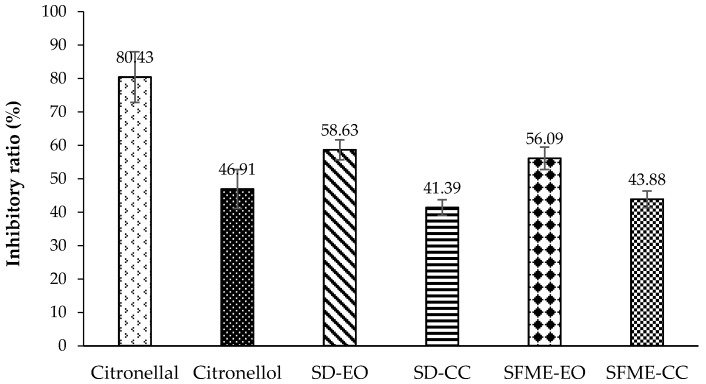
Tyrosinase inhibitory ratio (%) of ‘Mato Peiyu’ leaf essential oils, citronellal, and citronellol. SD-EO, SFME-EO, citronellal, and citronellol were measured at a concentration of 1.000% (*v*/*v*). SD-CC and SFME-CC were measured at concentrations of 0.507% and 0.598% (*v*/*v*) according to the composition ratios in 1.000% (*v*/*v*) SD-EO and SFME-EO, respectively. Data are presented as the mean ± standard deviation based on triplicate determination.

Tyrosinase is the rate-limiting enzyme responsible for melanin formation in the human skin [23,24]. The IC_50_ values of antityrosinase for ‘Mato Peiyu’ leaf SD-EO and SFME-EO were 0.771% and 0.882% (*v*/*v*), respectively (Table 5). SD-CC and SFME-CC exhibited similar inhibitory ratios of 41.39% and 43.88%, respectively (Figure 6). The other minor components of SD-EO demonstrated higher antityrosinase activity than that of SFME-EO, with 29.40% and 21.77% contributions, respectively (Table 5). SD-EO comprised a higher percentage of sesquiterpene than did SFME-EO (Table 1 and Table 2), such as β-caryophyllene, which possessed a higher antityrosinase activity [23]. Therefore, SD-EO exhibited a higher antityrosinase capacity than SFME-EO. Among all bioactivity assays, citronellal and citronellol exhibited lower antityrosinase activity than they did antimicrobial, antioxidant and anti-inflammation activities (Figure 1, Figure 2, Figure 3, Figure 4, Figure 5 and Figure 6). The antityrosinase inhibitory ratios of citronellal and citronellol were 46.91% and 80.43% (*v*/*v*), respectively, only when the concentration reached 1% (*v*/*v*) (Figure 6). The contribution of the SD-CC and SFME-CC on the antityrosinase ability of the essential oil was 70.60% and 78.23%, respectively (Table 4). This result implies that in addition to citronellal and citronellol, minor constituents in the essential oil exhibited some antityrosinase properties. Other studies have found that pomelo leaf essential oil from Mauritius was an effective tyrosinase inhibitor, with IC_50_ values of 2.07 ± 0.152 μg/mL, which is comparable to that of kojic acid [1]. However, ‘Mato Peiyu’ leaf essential oil exhibited weaker antityrosinase activity than it did antimicrobial (Table 3), antioxidant, and anti-inflammatory activities (Table 4). This is mainly because of the low antityrosinase capacity of the main components; additionally, the activities of minor constituents were too weak to provide a synergistic effect in the essential oil.

## 3. Materials and Methods

### 3.1. Chemicals and Reagents

DPPH, β-carotene, linoleic acid, chloroform, 5-LOX, α-tocopherol, Griess reagent, glycerol mono-oleate, sodium nitroprusside, sodium sulfate, 95% ethanol, Tween20, and Tween40 were purchased from Sigma-Aldrich (St. Louis, MO, USA). Methanol, l-ascorbic acid, 99% sodium chloride, and sodium phosphate dibasic were purchased from J.T. Baker (Phillipsburg, NJ, USA). l-Tyrosine and tyrosinase were purchased from Worthington (Lakewood, NJ, USA). 96% α-Bisabolol, 96% citronellal, and 95% citronellol were purchased From Alfa Aesar (Heysham, UK). Modified letheen broth was purchased from Oxoid (Basingstoke, UK). Agar, yeast extract, malt extract, peptone, and malt extract broth were purchased from HiMedia (Mumbai, India). Dextrose anhydrous was purchased from Nihon Shiyaku Reagent (Tokyo, Japan). Deionized distilled water was purified by the Milli-Q system (Millipore, Bedford, MA, USA).

### 3.2. Microorganisms

The microorganisms *S. aureus* ATCC 6538, *P. aeruginosa* ATCC 9027, *E. coli* ATCC 25922, and *C. albicans* ATCC 10231 were purchased from the Bioresource Collection and Research Center (Hsinchu, Taiwan). *E. coli*, *P. aeruginosa*, and *S. aureus* were incubated at 37 °C in TSB. *C. albicans* was cultured at 30 °C in YMPD medium (0.3% yeast extract, 0.3% malt extract, 0.5% peptone, and 1% dextrose).

### 3.3. Plant Materials

‘Mato Peiyu’ leaves were collected from an orchard (No. 41–6, Liaozibu, Madou District, Tainan City, Taiwan) in December 2016. The leaves were washed with tap water and were subsequently air-dried at room temperature for 3 days.

### 3.4. Preparation of Essential Oils

The dried-leaf sections underwent SD or SFME. SD was conducted using a modified Clevenger-type apparatus at 100 °C for 4 h. SFME was performed using a modified microwave machine (Sampo, Taoyuan, Taiwan) combined with a Clevenger-type apparatus. Before SFME, the dried leaves were soaked in water at room temperature for 1 h. The moistened leaves were subsequently heated in the SFME apparatus at 540 W for 40 min (10 min at less than 95 °C followed by 30 min at 95 °C). The distillates of the essential oils were subsequently separated, dried with anhydrous magnesium sulfate, filtered with a 0.45-mm filter, collected in amber vials, and maintained at 4 °C until further analysis.

### 3.5. Determination of Essential Oil Yield

The moisture content of the leaves was measured using a moisture analyzer (Precisa XM 60, Precisa Instrument Ltd., Dietikon, Switzerland). The yield of essential oil was calculated on the basis of dry weight by using the following equation:

Yield of essential oil (%) = (weight of essential oil/dry weight of material) × 100%(1)

### 3.6. Gas Chromatography–Mass Spectrometry Analysis of Essential Oils

The essential oils were diluted 1:99 (*v*/*v*) in methanol and analyzed for their chemical composition through GC–MS using an Agilent 7890A/5975MSD system (Agilent Technologies, Inc., Santa Clara, CA, USA) equipped with a HP-5MS capillary column (30 m × 0.25 mm i.d × 0.25 μm film thickness). The carrier gas, helium, was injected at a constant flow rate of 1.0 mL/min. The injector temperature was 250 °C. The sample (1 μL) was injected in the split mode (1:25). The oven temperature was initially 60 °C, was subsequently raised to 280 °C at 3 °C/min, and was then maintained at 280 °C for 70 min. The mass spectrometer was operated at an ionization voltage of 70 eV and a mass range of 35–400 *m*/*z*. The chemical constituents were identified by matching their retention time locking to *n*-pentadecane (at 27.500 min) and mass spectra with those obtained from the Flavor2.L, Wiley7n.1, and NIST98.L mass spectrum databases. The relative percentage of individual constituents was calculated on the basis of the gas chromatography peak area.

### 3.7. Determination of Bioactivities of Complete Essential Oils and Their Main Components

The antimicrobial, antioxidant, anti-inflammatory, and antityrosinase activities of the essential oils and their main components (individual and mixture) were measured as described in the following sections. The tested concentration of the main component mixture was the sum of the individual component concentration, which was calculated as the tested essential oil concentration multiplied by the relative percentage of individual component in the essential oil. Moreover, the effect of the main component mixture on the activity of the EO was quantified as the contribution and calculated using the following formula:

Contribution (%) = (Act_CC_/Act_EO_) × 100% (2)

where Act_CC_ and Act_EO_ are the activities of the main component mixture and essential oil, respectively.

#### 3.7.1. Determination of Antimicrobial Activity

The minimal inhibitory concentration values were determined using a broth dilution method developed by Rasooli et al. with modifications [74]. Serial dilutions of the essential oils were prepared in order to generate the concentrations of 0.010%, 0.025%, 0.050%, 0.075%, 0.100%, 0.150% and 0.200% (*v*/*v*) in corresponding media supplemented with Tween80 (0.5%) [63]. Fifty-microliter broth cultures of the various strains (1 × 10^7^ colony forming units [CFU]/mL) incubated overnight were inoculated into the various dilutions of the essential oils to obtain a final volume of 5 mL. After a 24-h incubation period, the culture broth was diluted 10-fold with modified letheen broth, and 100 μL of the cultures was spread on medium agar plates in triplicate. Plates were incubated for 24 h. The numbers of CFUs were counted and inhibitory ratios were calculated as follows:

Inhibitory ratios (%) = [(CFU_blank_ − CFU_sample_)/CFU_blank_] × 100% (3)

MIC_90_ was defined as the concentration of an essential oil necessary to inhibit 90% of the tested strain growth. The given concentrations of the main components were measured using methods identical to those used for the essential oils.

#### 3.7.2. Determination of Antioxidant Activity through DPPH Radical Scavenging Assay

The DPPH scavenging effect of the essential oils and the main compounds was determined using previously described methods with slight modifications [63,75,76]. A volume of 0.25 mL of the ethanolic solution of essential oils at various concentrations was individually mixed with 0.75 mL of ethanolic solution containing DPPH radicals (0.1 mM) to yield a final volume of 1 mL. The mixture was shaken vigorously and was placed in the dark for 30 min at 25 °C, and the absorbance was measured at 517 nm. A blank experiment containing all reagents except the test compound was conducted on the basis of the same procedure. α-Tocopherol was also analyzed as a reference. Tests were conducted in triplicate. The given concentrations of the main components were measured using methods identical to those used for the essential oils. DPPH radical scavenging activity was calculated using the following formula:

DPPH radical scavenging ratio (%) = [(Abs_blank_ − Abs_sample_)/Abs_blank_] × 100% (4)

where Abs_blank_ is the absorbance of the blank and Abs_sample_ is the absorbance of sample.

#### 3.7.3. Determination of Antioxidant Activity through β-Carotene/Linoleic Acid Assay

The β-carotene/linoleic acid assay was performed using previous described methods [63,77]. β-Carotene was dissolved in 0.2 mL of chloroform (1 mg/mL) and subsequently added to an emulsified mixture of linoleic acid (20 mg) in 200 mg of Tween20. Chloroform was evaporated in a vacuum at 40 °C for 5 min, and 50 mL of oxygenated distilled water was slowly added to the residue. The mixture was vigorously shaken to form an emulsion. A volume of 0.2 mL of various concentrations of the essential oils prepared in ethanol was added individually to 4.8 mL of the emulsion and subsequently incubated at 50 °C. The absorbance was measured at 470 nm after 3 h of incubation. A blank experiment containing all reagents except the test compound was measured at 470 nm immediately (0 h) and after 3 h of incubation. α-Tocopherol was also analyzed as a reference. Tests were conducted in triplicate. The given concentrations of the main components were measured using methods identical to those used for the essential oils. The antioxidant activity was evaluated using the following formula:

Inhibitory ratio of β-carotene/linoleic acid oxidation (%) = [(Abs_sample 3h_ − Abs_blank 3h_)/(Abs_blank 0h_ − Abs_blank 3h_)] × 100% (5)

where Abs_sample 3h_ is the absorbance of the sample at 3 h, Abs_blank 3h_ is the absorbance of the blank at 3 h, and Abs_blank 0h_ is the absorbance of the blank at 0 h.

#### 3.7.4. Determination of Antioxidant Activity through Nitric Oxide Scavenging Assay

To determine NO radical scavenging activity, the methods described by Marcocci et al. [78] and Tsai et al. [63] were employed. Various concentrations of the essential oils dissolved in ethanol were mixed individually with 0.1 mL of Tween20 and 0.1 mL of sodium nitroprusside (100 mM) in phosphate buffer solution (pH 7.4), and additional phosphate buffer solution (pH 7.4) was added to yield a final volume of 1 mL. After 2.5 h of incubation at 20 °C, 0.25 mL of the mixture was added to 0.25 mL of Griess reagent and reacted for 10 min at 25 °C. The absorbance was determined at 540 nm. A blank experiment containing all reagents except the test compound was conducted on the basis of the same procedure. α-Tocopherol was also analyzed as a reference. Tests were conducted in triplicate. The given concentrations of the main components were measured using methods identical to those used for the essential oils. NO radical scavenging activity was calculated using the following equation:

NO radical scavenging ratio (%) = [(Abs_blank_ − Abs_sample_)/Abs_blank_] × 100% (6)

where Abs_blank_ is the absorbance of the blank and Abs_sample_ is the absorbance of sample.

#### 3.7.5. Determination of Anti-Inflammatory Activity through 5-Lipoxygenase Inhibitory Activity Assay

5-LOX inhibitory activity was measured using previously described methods with linoleic acid as a substrate [63,79]. A volume of 30 μL of various concentrations of essential oil dissolved in ethanol was mixed individually with 30 μL of 0.1 mM linoleic acid solution in potassium phosphate buffer (PPB) (0.1 M, pH 6.3) with Tween 20 (0.60%) and 500 μL of 5-LOX solution (9.44 U/mL) in PPB (0.1 M, pH 6.3), and additional PPB was added to obtain a final volume of 3 mL. The mixture was incubated at 25 °C for 10 min, and the absorbance was determined at 234 nm. A blank experiment containing all reagents except the test compound was conducted on the basis of the same procedure. α-Bisabolol was also analyzed as a reference. Tests were conducted in triplicate. The given concentrations of the main components were measured using methods identical to those used for the essential oils. The inhibitory activity was calculated using the following formula:

5-LOX inhibitory ratio (%) = [(Abs_blank_ − Abs_sample_)/Abs_blank_] × 100% (7)

where Abs_blank_ is the absorbance of the blank and Abs_sample_ is the absorbance of sample.

#### 3.7.6. Determination of Antipigmentation through Tyrosinase Inhibitory Activity Assay

The tyrosinase inhibitory activity assay was conducted as described by Khatib et al. with slight modifications [80]. A volume of 100 μL of various concentrations of essential oil dissolved in ethanol was mixed individually with 300 μL of Tween20, 595 μL of tyrosine solution (0.33 mM) in phosphate buffer (16 mM, pH 6.8), and 5 μL of tyrosinase solution (35 U) in phosphate buffer (16 mM, pH 6.8). The mixture was incubated at 37 °C for 1 h, and the absorbance was determined at 475 nm. l-ascorbic acid was used as the positive control. For the blank, phosphate buffer was added to the reaction mixture instead of the test sample. Tests were conducted in triplicate. The given concentrations of the main components were measured using methods identical to those used for the essential oils. The inhibitory activity was calculated using the following formula:

Tyrosinase inhibitory ratio (%) = [(Abs_blank_ − Abs_sample_)/Abs_blank_] × 100% (8)

where Abs_blank_ is the absorbance of the blank and Abs_sample_ is the absorbance of sample

### 3.8. Statistical Analysis

Experimental data were analyzed using Excel 2013 (Microsoft Inc., Redmond, WA, USA). The results were expressed as the mean ± standard deviation based on triplicate determination. Significant differences between samples were identified when the *p*-values were <0.05 in Student’s *t*-test.

## 4. Conclusions

This is the first study analyzing the composition and bioactivities of ‘Mato Peiyu’ pomelo leaf essential oil. The compositions of ‘Mato Peiyu’ leaf essential oil were evidently different from those of pomelo extracts reported in previous studies. ‘Mato Peiyu’ leaf essential oil exhibited antimicrobial, antioxidant, anti-inflammatory, and antipigmentation abilities, whose potency was determined by the main components, citronellal and citronellol. In most assays, the essential oil isolated through SD exhibited greater bioactivities compared with that obtained through SFME. This study revealed that ‘Mato Peiyu’ leaf essential oil exhibited the highest potency of antimicrobial and anti-inflammatory activities among all bioactivity tests, suggesting the potential application of this essential oil in skin care. However, further study is required to confirm the antimicrobial activity of this essential oil on a significant strain number of the same bacteria. In addition, the safety of this essential oil including cytotoxicity and skin irritation and allergy requires further investigation.

## Figures and Tables

**Table 1:** Chemical composition percentage ^a^ of ‘Mato Peiyu’ leaf essential oil determined through gas chromatography–mass spectrometry analysis.

RT (min)	Compounds	SD-EO	SFME-EO
5.702	(−)-α-pinene	0.33	0.66
6.761	sabinene	1.43	3.01
6.881	(−)-β-pinene	7.33	9.58
7.234	myrcene	0.50	–
7.546	*cis*-2-(2-pentenyl)-furan	0.09	0.28
7.867	carene	0.35	0.60
8.468	dipentene	1.08	1.93
8.757	*cis*-β-ocimene	0.38	0.45
9.149	*trans*-β-ocimene	6.80	–
9.158	α-ocimene	–	6.38
9.318	melonal	–	0.11
10.600	δ-terpinene	0.14	–
11.025	linalool	0.50	0.25
11.466	*cis*-rose oxide	–	0.16
12.861	isopulegol	0.29	–
13.254	citronellal	34.54	30.87
14.168	4-carvomenthenol	0.21	–
16.420	citronellol	16.17	28.95
21.631	citronellyl butyrate	10.00	5.70
24.292	β-caryophyllene	8.22	2.76
25.623	α-humulenene	0.89	0.35
26.737	(−)-germacrene D	0.77	–
27.371	3,7,11,11-tetramethyl bicyclo[8.1.0]2,6-undecadiene	3.43	0.75
27.868	α-farnesene	1.57	0.37
28.421	δ-cadinene	0.47	–
29.679	γ-elemene	0.43	–
30.481	spathulenol	0.44	1.02
30.681	caryophyllene oxide	0.88	2.21
48.550	phytol	1.65	0.42
Total identified (%):		98.89	96.81

^a^ Values are presented as percentage of peak areas. RT: retention time. –: not detected. SD-EO: the essential oil obtained through steam distillation. SFME-EO: the essential oil obtained through solvent-free microwave extraction.

**Table 2:** Percentage ^a^ of various chemical classes of compounds in ‘Mato Peiyu’ leaf essential oil.

Chemical Class	SD-EO	SFME-EO
monoterpenes	18.34	22.61
sesquiterpenes	15.78	4.23
monoterpene alcohols	17.17	29.20
sesquiterpene alcohols	0.44	1.02
diterpene alcohols	1.65	0.42
monoterpene aldehydes	34.54	30.87
aldehydes	–	0.11
monoterpene esters	10.00	5.70
sesquiterpene epoxides	0.88	2.21
monoterpene cyclic ethers	–	0.16
furans	0.09	0.28
Total percentage:	98.89	96.81

^a^ Values are presented as total percentage of peak areas of compounds in the same chemical class. –: not detected.

**Table 3:** Minimum inhibitory concentrations (MIC_90_) (%, *v*/*v*) for the antimicrobial activity of ‘Mato Peiyu’ leaf essential oils.

Strains	SD-EO	SFME-EO
*S. aureus*	0.086 ± 0.001 ^aA^	0.098 ± 0.001 ^bA^
*P. aeruginosa*	0.103 ± 0.003 ^aB^	0.088 ± 0.005 ^bB^
*E. coli*	0.090 ± 0.001 ^aC^	0.116 ± 0.005 ^bC^
*C. albicans*	0.116 ± 0.004 ^aD^	0.121 ± 0.007 ^aC^

MIC_90_ refers to the minimum concentration required to inhibit 90% of the growth of strains. Data are presented as the mean ± standard deviation based on triplicate determination. Means in the same row followed by different lowercase letters are significantly different (*p* < 0.05). Means in the same column followed by different uppercase letters are significantly different (*p* < 0.05).

**Table 4:** Contribution (%) ^a^ of mixtures of citronellal and citronellol to the bioactivity of the essential oil.

Bioactivity	SD-CC	SFME-CC
Anti-*S. aureus*	66.46 ± 1.81	91.37 ± 7.99
Anti-*P. aeruginosa*	70.86 ± 4.56	60.89 ± 2.58
Anti-*E. coli*	54.58 ± 6.75	85.91 ± 4.44
Anti-*C. albicans*	126.17 ± 4.43	91.98 ± 6.45
DPPH radical scavenging activity	100.40 ± 0.21	115.06 ± 2.39
β-Carotene/linoleic acid inhibitory activity	81.45 ± 0.01	73.15 ± 0.60
NO radical scavenging activity	88.73 ± 6.28	95.60 ± 6.33
5-LOX inhibitory activity	71.92 ± 0.29	86.47 ± 8.18
Tyrosinase inhibitory activity	70.60 ± 4.03	78.23 ± 4.45

^a^ Contribution (%): the values from the bioactivity assays of the SD-CC and SFME-CC relative to the values from the bioactivity assays of the SD-EO and SFME-EO, respectively. Data are presented as the mean ± standard deviation based on triplicate determination.

**Table 5:** Scavenging concentration (SC_50_) (%, *v*/*v*) and inhibitory concentration (IC_50_) (%, *v*/*v*) for the antioxidant, anti-inflammatory, and antityrosinase activities of ‘Mato Peiyu’ leaf essential oil.

Scavenging Activity/Inhibitory Activity	SD-EO	SFME-EO	Tocopherol	Bisabolol	Ascorbic Acid
DPPH radical	0.752 ± 0.025 ^a^	0.876 ± 0.061 ^b^	0.0004 ± 0.0000 ^c^	-	-
β-Carotene/linoleic acid	0.147 ± 0.011 ^a^	0.086 ± 0.005 ^b^	0.060 ± 0.001 ^c^	-	-
NO radical	0.349 ± 0.010 ^a^	0.334 ± 0.003 ^a^	0.105 ± 0.005 ^b^	-	-
5-LOX	0.039 ± 0.004 ^a^	0.034 ± 0.001 ^a^	-	0.008 ± 0.000 ^b^	-
Tyrosinase	0.771 ± 0.056 ^a^	0.882 ± 0.056 ^b^	-	-	0.041 ± 0.000 ^c^

SC_50_: the concentration of a sample required for scavenging 50% of the free radicals in the diphenyl-1-picrylhydrazyl (DPPH) radical and nitric oxide (NO) radical scavenging assays. IC_50_: the concentration of a sample that resulted in 50% inhibition of the bioactivity in the β-carotene-linoleic acid, 5-lipoxygenase (5-LOX), and tyrosinase inhibitory activity assays. α-Tocopherol, β-bisabolol, and l-ascorbic acid were positive controls. Data are presented as the mean ± standard deviation based on triplicate determination. Means in the same row followed by different lowercase letters are significantly different (*p* < 0.05).
